# A Simple Approach to Characterize Gas-Aqueous Liquid Two-phase Flow Configuration Based on Discrete Solid-Liquid Contact Electrification

**DOI:** 10.1038/srep15172

**Published:** 2015-10-14

**Authors:** Dongwhi Choi, Donghyeon Lee, Dong Sung Kim

**Affiliations:** 1 Department of Mechanical Engineering, Pohang University of Science and Technology (POSTECH), 77 Cheongam-ro, Pohang, Gyeongbuk, 790-784, South Korea

## Abstract

In this study, we first suggest a simple approach to characterize configuration of gas-aqueous liquid two–phase flow based on discrete solid-liquid contact electrification, which is a newly defined concept as a sequential process of solid-liquid contact and successive detachment of the contact liquid from the solid surface. This approach exhibits several advantages such as simple operation, precise measurement, and cost-effectiveness. By using electric potential that is spontaneously generated by discrete solid–liquid contact electrification, the configurations of the gas-aqueous liquid two-phase flow such as size of a gas slug and flow rate are precisely characterized. According to the experimental and numerical analyses on parameters that affect electric potential, gas slugs have been verified to behave similarly to point electric charges when the measuring point of the electric potential is far enough from the gas slug. In addition, the configuration of the gas-aqueous liquid two-phase microfluidic system with multiple gas slugs is also characterized by using the presented approach. For a proof-of-concept demonstration of using the proposed approach in a self-triggered sensor, a gas slug detector with a counter system is developed to show its practicality and applicability.

A two-phase microfluidic system that requires two immiscible fluid phases inside a microfluidic channel has been rapidly developed in various science and engineering fields because of its own benefits, such as decreased sample/reagent consumption, reduced diffusion times, enhanced throughput, and fast response time^1^,^2^,^3^,^4^,^5^. In particular, gas–aqueous liquid two–phase flow plays an important role in a range of applications including information processing, micro heat exchanger, and lab-on-a-chip systems where mixing two fluids to prepare for further treatment is frequently desirable to obtain certain chemical reactions or to perform chemical analysis^6^,^7^,^8^,^9^. Given that the characteristics (length, volume, number, flow rate, and shape) of a segmented gas slug significantly affect to the enhancement of mixing, mass transfer, and heat transfer rate of the microfluidic system, the characterization of a gas slug in a two-phase flow has become significant to improve the performance of devices^3^,^10^,^11^,^12^,^13^,^14^,^15^,^16^. Moreover, it is also important in the biological and industrial systems to understand the reaction kinetics^17^. Since the lots of organic reactions such as hydrogenation of alkenes and Sandmeyer reactions generate products in the gas phase, the information relating to the reaction can be obtained by the quantification of gas slug/plug inside the microfluidic channel^17^. Considering that the dimensions of a microfluidic channel are approximately tens to hundreds of micrometers, investigating and characterizing a two-phase flow inside a microfluidic channel are crucial but challenging problems in this field.

To characterize configuration of gas-aqueous liquid two-phase flow (especially a gas slug) several techniques have been previously developed^13^,^17^,^18^,^19^,^20^,^21^. The available characterization strategies are mostly based on measuring the distorted laser light path attributed to the change in refractive index as a result of the presence of gas slugs^17^,^18^. In the pulse displacement technique, gas slug is detected and size is investigated through laser illumination and measurement of refracted and reflected beam paths^19^,^20^,^21^. Applying a similar concept, a real-time monitoring system in microfluidic channels that uses the difference between the refractive indices of the gas slug and the liquid has also been developed^19^. The incident beam of the laser is refracted by allowing to pass through the gas slug, which results in the difference in the light path. All aforementioned optics-based approaches are extremely sensitive to the alignment of devices. They also exhibit limitations, such as the constraint on the optical properties of channel materials (e.g., transparency and refractive index), because such properties considerably affect the light path through the microfluidic channel. In addition, expensive experimental equipment such as laser, power supply, camera, focusing lenses, and photodetector are required for accurately detecting and measuring gas slugs inside microfluidic channels.

Our group recently reported the spontaneous electrical charging of aqueous droplets through conventional pipetting, which was mainly attributed to solid–liquid contact electrification^22^,^23^,^24^. When a solid surface (i.e., the inner surface of a pipette tip) comes in contact with an aqueous liquid, electric charge is spontaneously generated on the contact surface; this phenomenon is called solid-liquid contact electrification. After solid-liquid contact electrification occurs, removing the liquid from the contact surface causes charge separation between the liquid and the solid surface, which makes net electric charge on the solid surface generating electric field and potential. In the present study, a sequential process of solid–liquid contact and successive detachment of the contact liquid from the solid surface is newly termed as a *discrete solid–liquid contact electrification* process resulting in generation of electric field and potential. In a gas-aqueous liquid two-phase flow inside a microfluidic channel, the presence of a gas slug plays a role in detaching liquid from the channel substrate, and thus, a discrete solid-liquid contact electrification process arises. As a result, electric potential is spontaneously generated by the net charge on the gas–solid contact surface in the microfluidic channel based on the same mechanism of the spontaneous charging of an aqueous droplet that detaches from the inner surface of the pipette tip^22^.

In the current work, a simple approach to characterize configuration of gas-aqueous liquid two–phase flow inside a microfluidic channel is first suggested and demonstrated. Given that the net electric charge is spontaneously generated on the microfluidic channel substrate as a gas slug passes through the channel, measuring the spontaneously generated electric potential caused by the electric charge on the substrate with the gas slug can be a simple indirect approach for characterizing the flow configuration. To measure the generated electric potential, we simply have to place the microfluidic chip on an additional electrode surface that is connected to an electrometer. Experimental and numerical investigations on the parameters that affect the amount of electric potential are systematically performed, and consequently, the gas slug inside the microfluidic channel is verified to behave similar to that of a point electric charge source when the measuring point of the electric potential is far enough from the gas slug. The average flow velocity inside the microfluidic channel is also demonstrated to be measured precisely by the proposed approach. In addition, the configuration of the gas-aqueous liquid two–phase microfluidic system with multiple gas slugs is characterized by using the presented strategy. Furthermore, for a proof-of-concept demonstration of the use of this strategy in a self-triggered sensor, a gas slug detector with a counter system is developed. It exhibits the practicality and applicability of the proposed approach. Given that this pioneering approach enables easy characterization of two-phase flow configuration by simply introducing an additional electrode to the conventional microfluidic chip, it exhibits several advantages including simple operation, precise measurement, self-triggering, and cost-effectiveness.

## Results And Discussion

### Basic principle to characterize gas-aqueous liquid two-phase flow configuration

**Figure 1:**
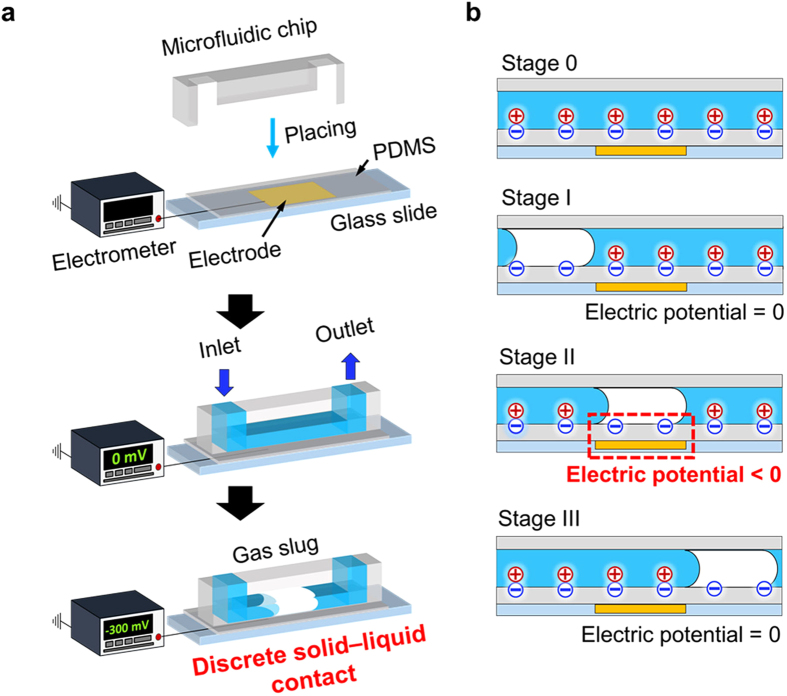
Schematic of the characterization strategy of gas-aqueous liquid two-phase flow configuration and the mechanism of electric potential generation caused by gas slug movement. (**a**) Schematic of the experimental setup for characterizing configuration of gas–aqueous liquid two-phase flow inside a microfluidic channel achieving discrete solid-liquid contact. (**b**) Mechanism of electric potential generated by discrete solid-liquid contact electrification inside a microfluidic channel from the movement of a gas slug.

Figure 1a shows schematic of the experimental setup and the procedure for characterizing flow configuration in gas-aqueous liquid two-phase microfluidic systems. The experimental setup consists of two simple steps. First, polydimethylsiloxane (PDMS) is spin-coated on an electrode-deposited glass slide, wherein the electrode is connected to an electrometer to measure the electric potential generated by the net electric charge on the solid substrate surface. Second, a microfluidic chip that includes an open channel is set on the PDMS–coated glass slide (Fig. 1a). Notably, the electrode-deposited glass slide can be reused to measure the electric potential because the electrode does not have contact with any liquid.

Given the experimental setup, we simply need to generate the gas-aqueous liquid two-phase flow inside the microfluidic channel, and then read the developed electric potential through the connected electrometer. The detailed procedure is as follows. Filling the microfluidic channel with aqueous liquid spontaneously generates an electric double layer (EDL) that consists of electric charge pairs. In this case, generating the electric charge pairs and the EDL is strongly affected by the zeta potential (*ζ*) of the solid surface with respect to the contact liquid. Numerous polymers have negative *ζ* relative to an aqueous liquid in nearly all experimental conditions, which results in a negative electric charge accumulation on the solid surface and a positive electric charge accumulation on the liquid side, as shown in Stage 0 of Fig. 1b. In this situation, no electric potential on the external electrode is measured because of electrical neutrality. When introducing a gas slug, a new gas–solid contact is formed, and consequently, a discrete solid–liquid contact electrification process occurs through successive gas slug movements. Gas slug movement sweeps away positive electric charges with liquid on the liquid side, and the concomitant detachment of positive electric charges generates a net negative electric charge in the gas–solid contact region of the solid surface, as illustrated in Stage I of Fig. 1b. Until this stage, electric potential on the external electrode does not change remarkably because the distance from the net negative electric charges on the solid surface to the electrode is too far to measure. Meanwhile, the positive charges on the discretized liquid side above the electrode are expected to effectively screen the electric field generated by the net charge. As the gas slug moves, a negative electric potential develops and the gas–solid contact region overlaps the external electrode region, as illustrated in Stage II of Fig. 1b. As the gas slug passes over the electrode region, electric potential continuously decreases (increases) as the overlapping region increases (decreases). After passing the electrode region, as shown in Stage III of Fig. 1b, the liquid following the gas slug comes in contact again with the solid surface. The net negative electric charge on the surface is rescreened by the positive charges in the liquid, and the measured negative electric potential disappears. According to the aforementioned mechanism of the electric potential development, the amount and behavior of the electric potential mainly depend on the characteristics of the gas slug. Consequently, the gas slug inside the microfluidic channel and the configuration of the gas-liquid two-phase flow can be indirectly characterized by simply measuring the developed electric potential on the substrate during gas slug movement over the electrode.

### Real-time measurement of the electric potential and parameters that affect the amount of electric potential

**Figure 2:**
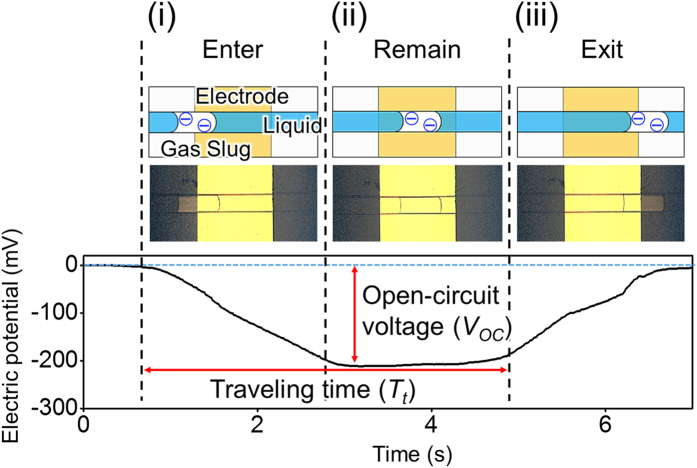
Representative images of the experimental result and the concomitant electric potential variation behavior. (**A**) U-shaped electric potential behavior appears with the movement of gas slugs within the microfluidic channel. The extreme value of the electric potential is designated as the open-circuit voltage (*V*_*OC*_). Traveling time (*T*_*t*_) is the moving time of a gas slug head from one end of the electrode to the other.

Figure 2 shows representative images of gas slug movement inside the microfluidic channel over the external electrode and the corresponding experimental result of the variation in the measured electric potential, which is a U-shaped electric potential variation as expected from the gas slug movement (Supplementary Video S1). Two important parameters are included in the plot: 1) the open-circuit voltage (*V*_*OC*_), which is a minimum plateau value of the measured electric potential that reflects the total net electric charge on the solid surface; and 2) traveling time (*T*_*t*_), which represents the flow time of the gas slug head from one end of the electrode to the other end; it implies the time-dependent behavior of the gas slug movement above the electrode. This electric potential behavior is similar to the result of the simplified numerical analysis of the moving gas slug inside the microfluidic channel with the ad hoc negative surface charge. (Supplementary Figure S1 and S2)

**Figure 3:**
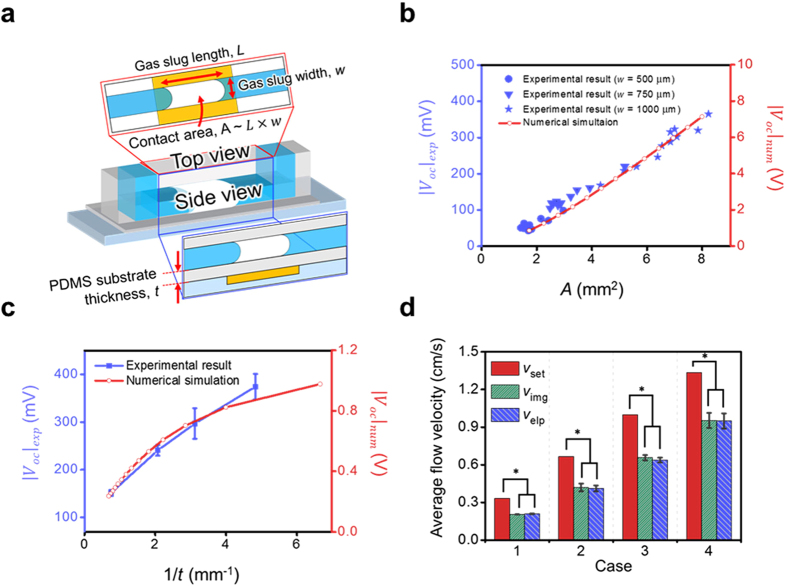
Parameters that affect the amount of electric potential and the demonstration of evaluating the average flow velocity within the PDMS microfluidic channel. (**a**) Designation of dimensions relevant to gas slug characteristics. (**b**) The effect of the contact area between the gas slug and the PDMS surface. The contact area (*A*) is linearly proportional to the amount of the open circuit voltage (*V*_*OC*_). (**c**) Effect of PDMS substrate thickness on the amount of the open-circuit voltage (*V*_*OC*_). The inverse of the substrate thickness is linearly proportional to the amount of the open-circuit voltage (*V*_*OC*_). (**d**) Demonstration of evaluating the average flow velocity within the PDMS microfluidic channel. *v*_*set*_ is calculated from the flow rate set on the syringe pump divided by the cross-sectional area of the microfluidic channel. Average flow velocities calculated from the captured images and measured from the electric potential behavior are denoted as *v*_*img*_ and *v*_*elp*_, respectively. At 5% significance level, significant differences exists between *v*_*set*_ and the other flow velocities.

Considering that the fundamental mechanism of the present characterization approach is a surface-related phenomenon, we investigate the behavior of *V*_*OC*_ when changing the contact area between the gas slug and the solid surface (*A*), where *A* ≈ *L* (length of a gas slug) × *w* (width of a gas slug), as shown in Fig. 3a. In Fig. 3b, the magnitude of *V*_*OC*_ is determined to be nearly linearly proportional to *A*, regardless of *w*. This relationship between *A* and *V*_*OC*_ is confirmed by the numerical analysis, although the magnitude of the electric potential is rather different. Since the aim of the numerical analysis is indirect verification of the fundamental mechanism of the present approach, our concern is the tendency of the generated electric potential, rather than the magnitude itself. The difference of the magnitude may arise from following reasons: 1) inaccurate surface charge density in the numerical analysis, 2) transformation of 3-dimensional empirical phenomenon into simplified 2-dimensional numerical domain (omission of the gas slug width, geometry of the channel and electrode). The result of numerical analysis enables us to suppose that the gas slug in our experiment is sufficiently small to be regarded as a point electric charge source rather than as a planar surface charge source. The electric potential developed at a distance *t* from the point electric charge source on the solid substrate to the external electrode, that is, the substrate thickness, in this case, can be expressed as follows:


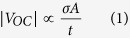


where *σ* is the surface charge density on the solid surface generated by discrete solid–liquid contact electrification. In Equation (1), an increase in *A* increases the total electric charge (*σA*) and thus strengthens the electric field at the electrode, which results in higher 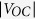. To verify Equation (1), the dependency of *t* on *V*_*OC*_ is also experimentally and numerically investigated by varying substrate thickness. As shown in Fig. 3c, a linearly proportional relationship exists between the inverse of the substrate thickness and the magnitude of *V*_*OC*_. However, it could be easily noticed that the slope of the electric potential becomes flatten as *t* becomes thinner. It indirectly shows that the gas slug behaves like a point electric charge source only when the measuring point of the electric potential is far enough (500 μm, in this experiment) from the gas slug. The detail result of numerical analysis about the thickness of the substrate to assume the gas slug as a point electric charge source is in the Supplementary Figure S3. Given that the microfluidic experiment is mostly performed with the sufficiently thick substrate such as a glass slide, the assumption of the point electric charge source is obviously reasonable in most of microfluidic experiments. Numerically and experimentally proven point electric charge source assumption enables to apply the concept of discrete solid-liquid contact electrification to the characterization of a gas-aqueous liquid two-phase flow in microfluidic system. Although it is verified that the present approach could be applied in the micro-system with the point electric charge source assumption, there might be difference between the micro-system and bulk-system. Given that width of microfluidic channel (same as the width of the gas slug, *w*) as well as *t* are important factors which determine the size of the system, we perform the experiment to investigate the dependency of *w* on *V*_*OC*_. As a result, it is checked that there is a critical width of the microfluidic channel which is a maximum width where the point electric charge source assumption in this study is valid. The detailed information about it is in the Supporting Information. In our system, it is verified that the critical width seems to exist within *w* range of 1500 ~ 2000 μm and thus, it indirectly shows that the point electric charge source assumption is valid with smaller *w* than the critical width (Supplementary Figure S4). Since width of the microfluidic channel is usually order of 100 μm, the result shows the present approach is valid in most of microfluidic experiments. However, for applying the present approach to the bulk-system, there is a need to perform further study about critical bulk factors such as width and thickness with comprehensively explored fundamental phenomenon of this approach.

Utilizing the electric potential behavior generated by the moving gas slug inside the microfluidic channel, the average flow velocity can also be measured. The time-dependent parameter (*T*_*t*_), which is illustrated in Fig. 2, of the electric potential in response to the moving gas slug enables the evaluation of the average flow velocity within the microfluidic channel. The length of the electrode (2 cm in our experiment) is known; therefore, average flow velocity can be calculated by dividing electrode length by *T*_*t*_. To demonstrate this procedure, the experiments with flow velocity (*v*_*set*_), which varies from 0.3 to 1.3 cm/s (where *v*_*set*_ is calculated from the flow rate set on the syringe pump divided by the cross-sectional area of the microfluidic channel), are performed as shown in Fig. 3d. The average flow velocities calculated from the captured images and the electric potential behavior are denoted as *v*_*img*_ and *v*_*elp*_, respectively. Among three variables, *v*_*img*_ is the most accurate and is analogous to the real movement of the gas slug because it directly presents the actual situation within the microfluidic channel. As shown in Fig. 3d, important differences clearly exist between *v*_*set*_ and *v*_*img*_, which indicates that the real flow velocity within the PDMS microfluidic channel differs from the flow velocity calculated using the flow rate set on the syringe pump. A number of studies have recently reported that inaccurate estimates of the average flow velocity within PDMS microfluidic channels have several causes, such as syringe pump-induced fluctuation, deformation of a flexible PDMS microfluidic channel and tubing system, and solvent-induced PDMS swelling behavior^25^,^26^,^27^. Thus, the enlargement of the cross-sectional area of the PDMS channel can explain the difference between *v*_*set*_ and *v*_*img*_, that is, *v*_*set*_ > *v*_*img*_. By contrast, no significant difference is observed between *v*_*elp*_ and *v*_*img*_. The experimental result implies that the average flow velocity that has been analyzed using the proposed approach based on discrete solid-liquid contact electrification (*v*_*elp*_) is more analogous to the real movement of the gas slug within the PDMS microfluidic channel (*v*_*img*_) than the flow velocity set on the syringe pump (*v*_*set*_). Consequently, the average flow velocity within the PDMS microfluidic channel can be conveniently and accurately evaluated with the presented approach.

### Characterization of the two-phase flow configuration with multiple gas slugs

**Figure 4:**
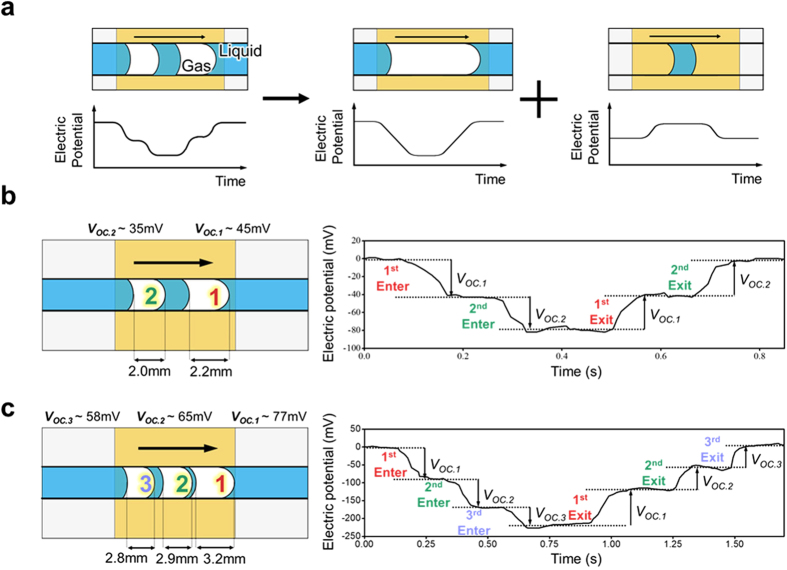
Evaluation of the two-phase flow configuration with multiple gas slugs. (**a**) The behavior for two gas slugs is expected to be the superposition of the two potential behaviors for the gas slug and liquid plug, which is represented as a two-step shape. (**b**) Experimental result obtained for two successive gas slugs. A two-step shape is observed as expected. (**c**) Experimental result obtained for three successive gas slugs, showing a three-step shape.

The electric potential behavior during the movement of multiple gas slugs is further investigated to identify more practical applications of the proposed approach to characterize a configuration of a two-phase flow with multiple gas slugs. As shown in Fig. 4a, two gas slugs can be decomposed into a single huge gas slug with a small liquid plug inside it without losing generality. Recalling the U-shaped variation in the electric potential in response to the movement of a single gas slug across the electrode (Fig. 2), the behavior of the electric potential in response to the movement of the liquid plug will be the reverse of that of the gas slug because liquid plays a role in removing net negative charge from the solid surface, and thus, an increase in electric potential will result. Consequently, the behavior during the movement of two gas slugs is expected to be a superposition of these two potential behaviors, which is represented as a two-step shape in Fig. 4a. In this case, the important point is the capability to evaluate a liquid plug in the continuous gas phase of a two-phase gas–liquid flow by observing the electric potential behavior, which has a “reverse U-shape”. That is, this novel characterization strategy will be used to characterize the movement of liquid and gas slugs using the same basic mechanism based on the proposed approach. The resultant potential behavior can also be explained by the verified assumption of the point electric charge. That is, given that multiple gas slugs can be regarded as separate multiple point electric charges, the resultant electric potential can be calculated as an arithmetic summation of each potential developed by each point electric charge.

To verify the electric potential behavior during the movement of multiple gas slugs, experiments using two and three gas slugs are performed. In Fig. 4b, two gas slugs with different lengths pass through the microfluidic channel over the electrode. As the first slug enters the electrode region, the measured electric potential starts to decrease with the increasing overlap between the gas slug and the electrode. In this case, the second slug enters the electrode region before the first slug leaves this region, and thus, electric potential further decreases. Consequently, a downward two-step shape of the electric potential is measured, as plotted in Fig. 4b, and the result is as expected. The two gas slugs consecutively leave the electrode region; hence, the electric potential behavior is an upward two-step shape. Similarly, the measured electric potential has a three-step shape when three gas slugs are present, as shown in Fig. 4c. Based on the results, we conclude that the electric potential behavior for *N* gas slugs would have an *N*-step shape with each step representing one gas slug (or liquid plug). This characterization of the configuration of two-phase flow with multiple gas slugs demonstrates that the present novel approach based on discrete solid–liquid contact electrification cannot only characterize a single gas slug, but also multiple gas slugs and even liquid plugs inside a microfluidic channel.

### Self-triggered gas slug sensor

**Figure 5:**
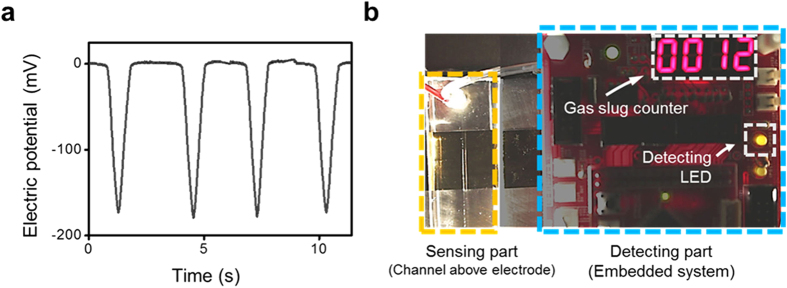
Demonstration of a self-triggered gas slug detector with a counter system, which counts the number of gas slugs passing over an electrode. (**a**) Electric potential behavior with multiple gas slugs consecutively passing over the electrode within a straight microfluidic channel. (**b**) The detecting part, which consists of a computer and an embedded system, is directly connected to the sensing part, which consists of a microfluidic channel and an electrode for real-time detection of gas slugs inside a microfluidic channel.

The present approach for characterizing two-phase flow configuration based on discrete solid–liquid contact electrification can be applied practically to the development of self-triggered sensors for sensing the gas slug within a continuous aqueous liquid flow inside the microfluidic channel through the generation of a voltage output (electric potential) during the movement of a gas slug. Several recent studies have reported on the use of solid–liquid contact electrification as an energy harvesting method and self-powered or triggered sensors that use the generated electric output^28^,^29^,^30^,^31^. As a proof-of-concept application of such sensor, a gas slug detector with a counter system, which counts the number of gas slugs passing over the electrode, is developed. When the gas slugs inside the straight microfluidic channel are consecutively passing over the electrode, the developed electric potential behavior on the electrode is shown in Fig. 5a according to the aforementioned principle. Given that the magnitude of the *V*_*OC*_ from the gas slug is well above the noise level, the presence of a gas slug is easy to be detected from the electric potential behavior. To achieve real-time detection of a gas slug, an additional detecting part, which consists of a computer and an embedded system with an external electric power, is directly connected to the sensing part, which consists of a microfluidic channel and an external electrode as shown in Fig. 5b (Supplementary Video S2). When a gas slug enters (exits) the electrode region, the electric potential starts to change rapidly. This sudden change in electric potential triggers the operation of the detecting part with an external electric power, which results in the counting of gas slugs. This proof-of-concept demonstration of the self-triggered gas slug sensor shows the practicality and applicability of the proposed simple and self-triggered characterizing strategy.

## Discussion

As the first demonstration, this study exploits a discrete solid-liquid contact electrification phenomenon to characterize configuration of gas-aqueous liquid two-phase flow inside a microfluidic channel. In this study, the connection between the spontaneously developed electric potential and the configuration of the two-phase flow allows simple indirect measurement of the size and moving velocity of the gas slug which have of paramount importance in various gas-aqueous liquid processing systems. Since the electric potential is generated by the net electric charge on the substrate, the necessary condition for measurement is contact between the gas slug and the solid substrate with concomitant generation of the net electric charge. In other words, it could not be applied on the two-phase systems including gas bubble passage (not gas slug phase), which does not contact with the substrate at all. Although this becomes major shortcoming of the proposed approach, this electric charge based approach has possibility to be utilized on the above system with the generally accepted concept that the gas/liquid interface also carries electric charges. Then, the applicability of the present approach could be strongly broaden to the wetting system with gas bubble passage. The further study about it is needed to clarify the applicability of the present method. Along with it, for applying the present approach to the bulk-system, there is also a need to perform comprehensive study about critical bulk factors such as width and thickness, as mentioned above.

There is another topic of interest with the present characterization approach; it is about other characteristics which could affect to *V*_*OC*_. Given that the fundamental mechanism of the present approach is based on generation of the net electrical charges on the solid substrate caused by discrete solid-liquid contact electrification, another crucial factor determining *V*_*OC*_is the amount of generated net electrical charges (related with *σ* in Equation (1)). The amount of generated net electrical charges on the substrate is highly affected by various aqueous liquid characteristics such as electrolyte concentration, pH, and temperature^22^,^23^,^24^,^29^. Although there is no intensive exploration about these characteristics in this study, the effect of them to the amount of net electrical charges could be mentioned here with lots of previous literatures^22^,^23^,^24^,^29^. According to the literatures, we could expect that higher electrolyte concentration, lower pH, and higher temperature of the aqueous liquid would generate lower *V*_*OC*_ with decreased amount of net electrical charges. Along with the liquid characteristics, the solid substrate characteristics could also affect to *V*_*OC*_. For example, in terms of surface energy of the solid substrate, it affects to the detachment of the aqueous liquid, which is quite important to the separation of electric charges (shown in Fig. 1b). In that sense, we could also expect that the lower surface energy, which means hydrophobic solid substrate, generates higher *V*_*OC*_ with increased amount of net electrical charges on the solid substrate. Consequently, aforementioned discussions represent that the further study about various characteristics of aqueous liquid and solid substrate, which affects to the amount of net electrical charges generated from discrete solid-liquid contact electrification, should be performed before to utilize the present approach with more practical applications.

In summary, we demonstrate a novel approach to characterize gas-aqueous liquid two-phase flow configuration based on *discrete solid–liquid contact electrification*. The characteristic of a gas slug in gas–aqueous liquid two-phase microfluidic systems is indirectly investigated using the spontaneously generated electric potential. Placing the conventional microfluidic chip onto the reusable electrode-deposited substrate enables easy measurement of the electric potential generated by the movement of gas slugs. Consequently, the U-shaped electric potential behavior is measured in response to the movement of gas slugs over the electrode region. The assumption that a gas slug inside the microfluidic channel behaves similar to that of a point electric charge is verified *via* the theoretical and experimental analyses. Furthermore, experiments on multiple gas slugs are performed and analyzed to demonstrate that the proposed novel characterizing approach based on discrete solid-liquid contact electrification can be used for a single gas slug, multiple gas slugs, and even a liquid plug in two-phase flows. For a proof-of-concept demonstration of the application of the self-triggered gas slug sensor, a gas slug detector with a counter system is developed. The present discrete solid-liquid contact electrification assisted approach, which is the first to apply to characterize, will provide a powerful characterization platform for gas–aqueous liquid two-phase microfluidic systems.

## Methods

### Fabrication of the device

The poly-dimethylsiloxane (PDMS) microfluidic chip for generating gas-liquid two-phase flow was fabricated using the standard soft-lithography technique. Briefly, a 150-μm-thick SU-8 2150 photoresist was spin-coated on a 4-inch silicon wafer. The SU-8 layer was selectively exposed to the UV light and then developed to obtain a master. The PDMS pre-polymer was mixed with a curing agent in a 10:1 weight ratio and degassed. After pouring the PDMS mixture over the fabricated master, it was cured at 65 °C for 4 hours, and then it was peeled off from the master. Along with, a metal electrode for measuring the electric potential was selectively deposited onto a conventional glass slide and the PDMS is directly spin-coated with the controlled thickness with an adequate rotational speed of the spin coater. The PDMS microfluidic chip including an open channel is set on the PDMS coated glass slide.

### Real-time measurement of electric potential

The aqueous liquid with gas slugs of desired volumes was introduced through tubing by using the syringe pumps (KDScientific KDS230). The inlet and outlet of the PDMS microfluidic chip were both electrically grounded to minimize the noise from the unexpected charge in fluid. The metal deposited on the glass slide was electrically connected with the electrometer (Keithley 6517A) for measuring the generated electric potential. The electrometer was synchronized with a CCD camera (Viewworks VH-310C). Both electric potential and image of the microfluidic channel were recorded on a computer in real time.

## Additional Information

**How to cite this article**: Choi, D. *et al.* A Simple Approach to Characterize Gas-Aqueous Liquid Two-phase Flow Configuration Based on Discrete Solid-Liquid Contact Electrification. *Sci. Rep.* **5**, 15172; doi: 10.1038/srep15172 (2015).

## Supplementary Material

Supplementary Information

Supplementary Video S1

Supplementary Video S2
