# Supplementary material for: A Simple Approach to Characterize Gas-Aqueous Liquid Two-phase Flow Configuration Based on Discrete Solid-Liquid Contact Electrification
Source: Sci Rep. 2015 Oct 14;5:15172. doi: 10.1038/srep15172 (PMC4604473; doi:10.1038/srep15172)
Supplement: Supplementary Information [file srep15172-s1.pdf]

Supplementary Information

A Simple Approach to Characterize Gas–Aqueous Liquid Two-phase Flow Configuration Based on Discrete Solid-Liquid Contact Electrification

Dongwhi Choi[#], Donghyeon Lee[#], and Dong Sung Kim[*]

Department of Mechanical Engineering, Pohang University of Science and Technology (POSTECH), 77 Cheongam-ro, Pohang, Gyeongbuk, 790-784, South Korea

[#]These authors contributed equally to this work.

[*]To whom all correspondence should be addressed.
Full postal address:
Prof. Dong Sung Kim
Department of Mechanical Engineering, Pohang University of Science and Technology (POSTECH), 77 Cheongam-ro, Pohang, Gyeongbuk, 790-784, South Korea
Email: smkds@postech.ac.kr

Numerical analysis of the system

For numerical analysis, the system of the gas slug inside the microfluidic channel with the additional external electrode for electric potential measurement is simplified as the numerical domain shown in Figure S1a. Since the gas slug inside the microfluidic channel could be treated as a net negative electric charge, the gas slug corresponds with the surface charge in the domain. To determine the amount of the surface charge density, the amount of the charge of the DI water droplet dispensed from the PDMS-coated capillary is measured by using the Faraday cup method. After simple calculation, the surface charge density is determined as -1.175×10^{-7}C/m^2. To represent the aqueous liquid around the gas slug, four imaginary lines are drawn. Since the aqueous liquid is all electrically grounded, the electrical ground boundary condition is applied. The sizes of the imaginary ground including length of the aqueous liquid are decided large enough to not affect to the electric potential generated by the gas slug as shown in Figure S1b and S1c. To check its possibility of use, the electric potential behavior caused by the moving gas slug is simulated by changing the relative distance between the gas slug and the electric potential measuring point, *r*, from -5 to 5 mm. As shown in Figure S2, the behavior is similar with the U-shaped electric potential behavior in the experimental result.

Thickness of the substrate to assume the gas slug as a point electric charge source

The dependency of t on V_{OC} is numerically investigated by varying substrate thickness. As shown in Figure S3a, the electric potential shows linearly proportional relationship with the inverse of the substrate thickness when the thickness is sufficiently thick (over 500 μm, in this experiment). However, as the substrate becomes thinner, the slope becomes flatten, which indirectly means that the gas slug behaves like a plane electric charge source rather than a point electric charge source. It could be simply explained with the equipotential lines around the gas slug as shown in Figure S3b. Although the equipotential lines which locate sufficiently far from

the gas slug show similar characteristics of the electric potential generated from the point electric charge source as plotted in Figure S3c, the lines nearby the gas slug follow the characteristic of the electric potential generated from the plane electric charge source in Figure S3d. This behavior mainly be attributed to the existence of the grounded aqueous liquids around the gas slug. The further study is needed to clarify the role of the grounded aqueous liquids around the gas slug.

Width of the microfluidic channel to assume the gas slug as a point electric charge source

The dependency of w on V_{OC} is experimentally investigated to explore the validity of the present approach to the bulk-system by varying w from 250 to 4000 μm with fixed t (= 500 μm). As shown in Figure S4a, V_{OC} is linearly proportional to w with range from 250 to 1500 μm. However, as the channel becomes broader, the increase behavior of V_{OC} becomes retarded compared to the proportional relationship. To deeply investigate such behavior, V_{OC} per contact area ($|V_{oc}|/A$) is calculated to ignore the effect of the gas slug contact area (A). The amount of $|V_{oc}|/A$ is maintained as constant at w range from 250 to 1500 μm as shown in Figure S4b. Although the experimental data in that range shows the validity of the point electric charge assumption, the remaining data at the higher w range implies that there exists in critical width which is a maximum width where the point electric charge source assumption is valid. In our experiment, the critical width seems to exist within the w range of 1500 ~ 2000 μm and thus, it indirectly shows that the point electric charge source assumption is invalid with higher w than the critical width. Consequently, for applying the present approach to the bulk-system, there is a need to perform further study about critical width of the microfluidic channel.

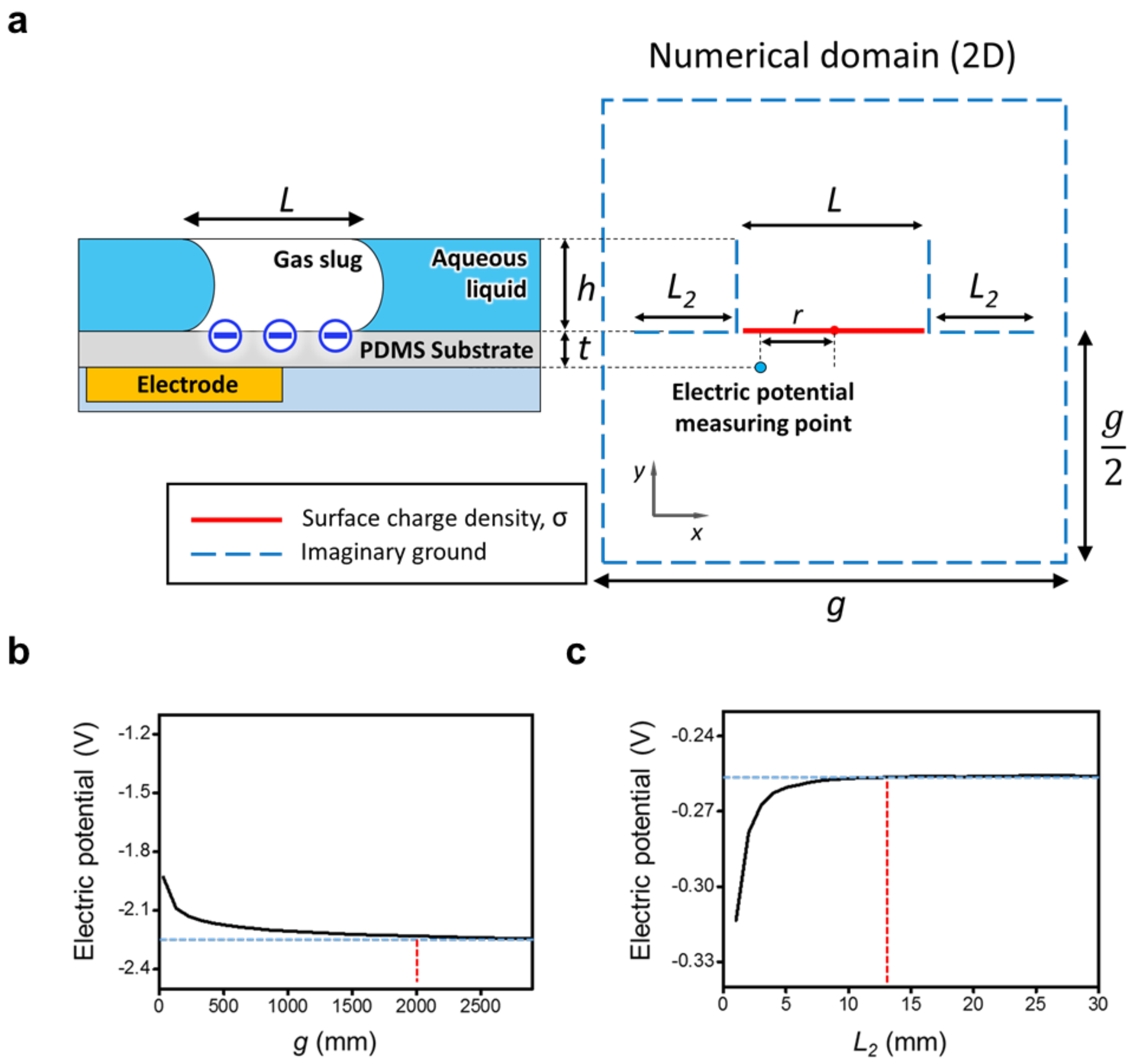

Figure S1 Schematic diagrams of the experiment and numerical analysis for the net charge on the microchannel substrate. (a) Domain of numerical analysis and applied boundary condition. (b) Determination of imaginary ground size, g. (c) Determination of imaginary aqueous liquid size, L_2.

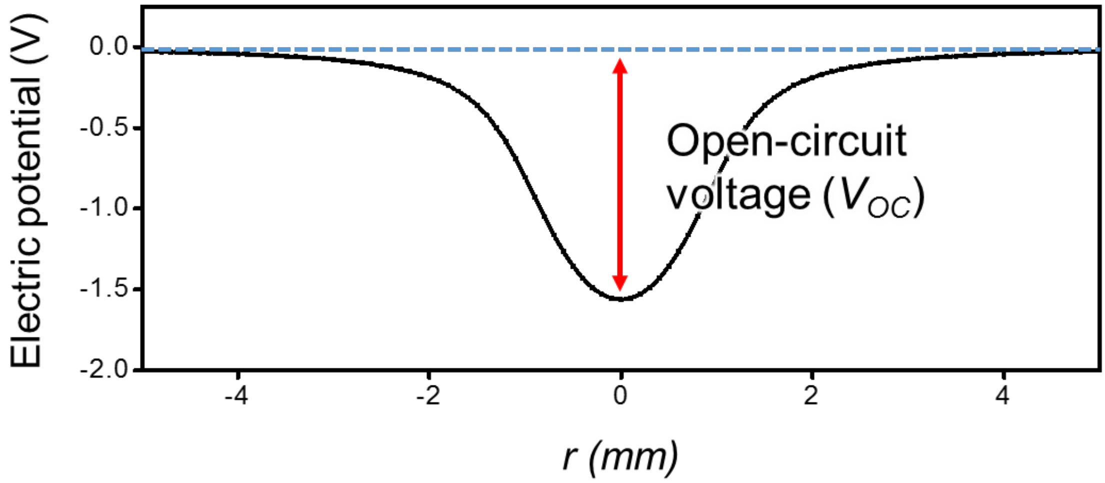

Figure S2 Numerical result of the electric field behavior generated by the surface electric charge with respect to the relative distance with the electric potential measuring point. Relative distance increases from -5 to 5 mm. The behavior is similar with the U-shaped electric potential behavior in the experimental result.

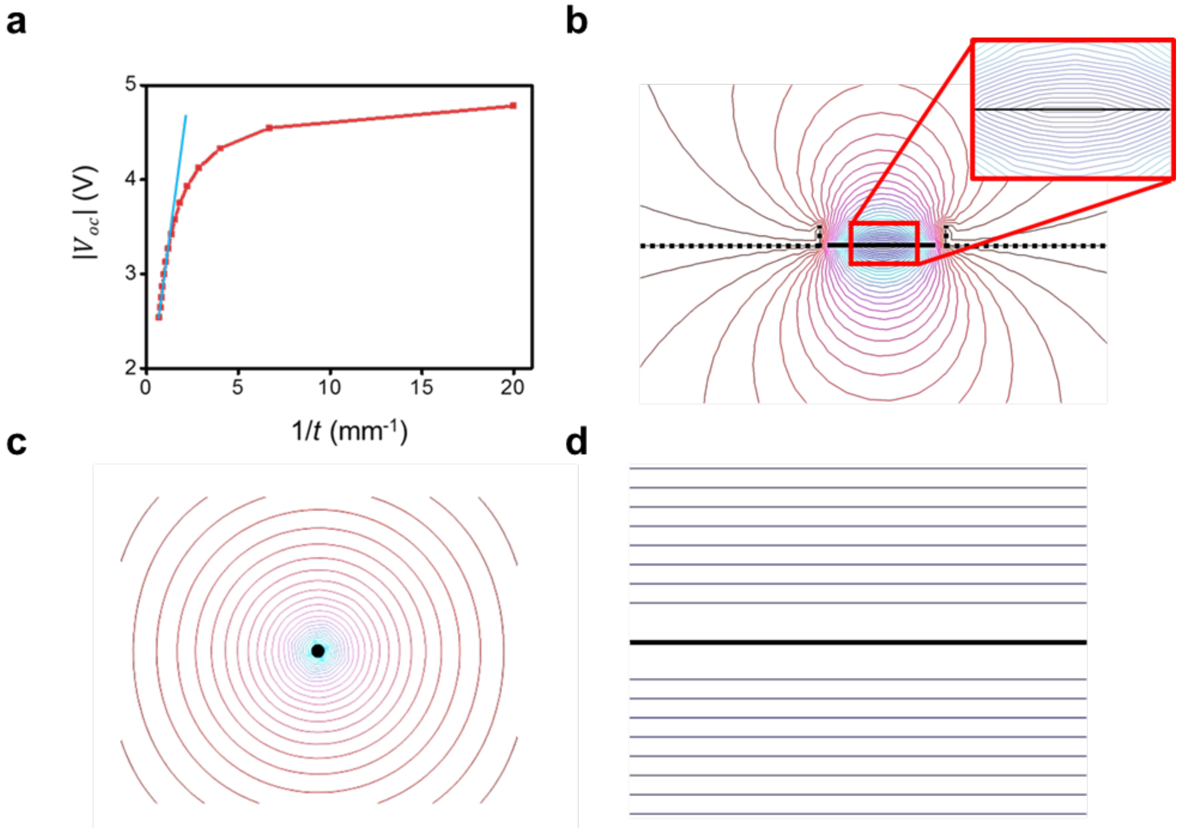

Figure S3 Numerical simulation results of the generated electric potential as a function of substrate thickness, *t*. (a) The electric potential is linearly proportional to the inverse of the solid substrate. As the substrate becomes thinner, the slope becomes flatten, which indirectly means that the gas slug behaves like a plane electric charge source rather than a point electric charge source. (b) Result of the numerical simulation regarding the electric potential around the gas slug. Each contour represents equipotential line. (c) Result of the numerical simulation regarding the electric potential around the point electric charge source. (d) Result of the numerical simulation regarding the electric potential around the plane electric charge source.

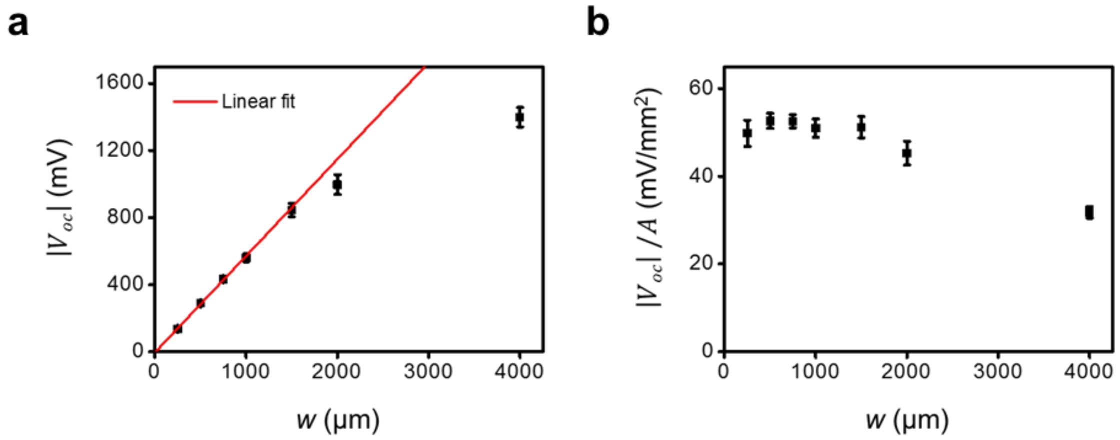

Figure S4 Measurement of V_{OC} with varying microfluidic channel width. (a) V_{OC} is linearly proportional to w with range from 250 to 1500 μm. However, the increase behavior of V_{OC} becomes retarded compared to the proportional relationship as w becomes larger. (b) V_{OC} per contact area ($|Voc|/A$) is calculated to ignore the effect of the gas slug contact area. The amount of $|Voc|/A$ is maintained as constant with some variation at w range from 250 to 1500 μm. The critical width seems to exist within the w range of 1500 ~ 2000 μm.
